# The diagnostic value of [^18^F]-FDG-PET/CT in assessment of radiation renal injury in Tibet minipigs model

**DOI:** 10.1186/s12967-018-1626-0

**Published:** 2018-09-17

**Authors:** Yu-Guang Tian, Min Yue, Bayaer Nashun, Shao-Jie Wu, Wei-Wang Gu, Yu-Jue Wang

**Affiliations:** 1 Department of Laboratory Animal Center, Southern Medical University, 1838# Guangzhou North Road, Guangzhou, 510515 Guangdong China; 2 Department of Guangdong Medical University, Dongguan, 523808 People’s Republic of China; 3 Department of Hemotology, Zhujiang Hospital, Southern Medical University, 253# Industry Road, Guangzhou, 510282 Guangdong China

**Keywords:** ^18^F-FDG PET/CT, Kidney, Total body irradiation, Tibet minipig, Radiation damage, Rapid assessment

## Abstract

**Background:**

Radiation-induced kidney damage can severely affect renal function, and have a serious impact on glucose reabsorption. Fluoro-2-deoxyglucose positron emission tomography (FDG-PET) is routinely utilized for metabolic imaging of glucose utilization. In this study, we are trying to assess the diagnostic value of ^18^F-FDG-PET/CT on measuring hyperacute effect of total body irradiation (TBI) on the kidneys.

**Methods:**

Forty-eight Tibet minipigs were treated by TBI of different dosages using an 8-MV X-ray linear accelerator. Whole-body ^18^F-FDG-PET/CT was performed at 6, 24 and 72 h followed by histologic examination, blood samples’ and renal function analysis.

**Results:**

The uptake of ^18^F-FDG was significantly different between 11/14 Gy dose groups and control group, the standard Uptake Values reached a maximal level at 72 h after 14-Gy TBI treatment. At doses over 8 Gy, histological observation showed formation of tube casts, degeneration, necrosis of tubular cells, inflammatory cell infiltration and dilatation of the mitochondria of tubule cells. Renal function analysis confirmed the changes in blood urea nitrogen and creatinine levels at various dosages and time intervals. Immunohistochemistry and western blot results indicate that the expression levels of IL-10 and TNF-α proteins were positively correlated with radiation dose up to 8 Gy.

**Conclusions:**

^18^F-FDG PET/CT can reflect pathological changes in kidneys and it may be a useful tool for rapid and non-invasive assessment in cases of suspected radiation-induced kidney damage.

## Background

Radiation-induced kidney damage (RIKD) can severely affect renal function, and have a serious impact on glucose reabsorption [1]. Fluoro-2-deoxyglucose positron emission tomography (FDG-PET) is routinely utilized for metabolic imaging of glucose utilization. We therefore presume that FDG-PET can be used to detect radiation-induced changes in renal function. Furthermore, our previous study showed in the Tibet minipig model, that radiation doses are strongly correlated with the uptake of 18-fluoro [^18^F]-FDG in the spleen and hematopoietic system [2, 3]. As is well known, kidney is one of the dose-limiting organs for radiotherapy and during total-body irradiation (TBI) [4]. They are also vitally important, being responsible for filtering waste metabolites and modulating blood pressure and electrolytes from the blood and the fluid/electrolyte balance, and producing erythropoietin to stimulate red blood cell production. At present, radiation damage can be detected in animal models by means of both noninvasive and invasive assays that are used to detect kidney injury in humans: e.g., measurement of blood urea nitrogen (BUN) [5] and creatinine (Cr) [6], the glomerular filtration rate (GFR) [7], histological examination, and diagnostic imaging techniques such as X-ray computed tomography (CT) and scintigraphy. Currently, combination of biochemical, scintigraphic and biopsy examinations is the gold standard for diagnosis of RIKD, although some limitations still exist. For example, the BUN value can be influenced by various factors such as an individual’s nutritional condition and concurrence of gastrointestinal hemorrhage. RIKD is not always accompanied by reduced glomerular function, which is estimated using the Cr value. The extent of scintigraphic changes can fluctuate depending on time after irradiation, radiation dose and irradiated volume. Biopsy is often more invasive than other examinations. We therefore set out to examine whether ^18^F-FDG-PET/CT can be used as a metabolic imaging technique and may have a role in the assessment of RIKD.

Previous studies have shown that Tibet minipigs are genetically stable and small, mature early, and have a high fecundity rate [8, 9]. Therefore, we selected the Tibet minipig as an animal model for the study of RIKD. ^18^F-FDG PET/CT is a noninvasive diagnostic technique utilizing biochemical metabolic differences between benign and malignant tissues. Its application has experienced explosive growth as a clinical modality. However, FDG is a marker of glycolysis and does not specifically accumulate in malignancy. Various degrees of FDG uptake are therefore present in normal tissues and are noted in benign processes [10]. No evidence has previously been presented concerning the hyperacute effect of irradiation on glucose metabolism using FDG in kidney tissue. In this study, we used a range of indices, such as FDG-PET/CT, BUN and pathologic examination to determine: (1) whether the change patterns of hepatic ^18^F-FDG uptake within 72 h post-TBI could be used for diagnostic purposes; (2) whether the [^18^F]-FDG-PET/CT could be used for diagnosing hyperacute TBI-induced kidney injury; and (3) how to apply this knowledge in a clinical setting.

## Methods

### Animals

Animals were housed in an Association for Assessment and Accreditation of Laboratory Animal Care (AAALAC) approved facility at the Southern Medical University Laboratory Animal Center (SMULAC). All procedures involving animals were reviewed and approved by the SMULAC Institutional Animal Care and Use Committee (IACUC) and all efforts were made to minimize suffering. Adult male Tibet minipigs (8–15 months, weighing 21.16 ± 5.54 kg, supplied by the Center of Laboratory Animal, Southern Medical University) were kept under standard laboratory conditions with a 12 h light and 12 h dark cycle and allowed to take food and water freely. This study was approved by the supervising state agency (License Number: scxk Yue 2006-0015) and performed in full accordance with the state guidelines.

### Radiation protocol

Forty-eight anesthetized (0.15 mL/kg SuMianXin II, compound of ethylenediamine tetraacetic acid (EDTA), hydrochloric dihydroetorphine and haloperidol, purchased from the Military Veterinary Institute, Quartermaster University) Tibet minipigs were divided into one control group and five treatment groups and placed in a platform for fixation postures and subjected to irradiation exposure by an 8MV X-ray (isocenter) linear accelerator (Precise System Treatment System, Elekta, Sweden). The irradiation was carried out at the Cancer Centers of Armed Police Hospital of Guangdong as described in another study (14). Animals in the control group (n = 3) were not exposed to X-ray irradiation, while those in the treatment groups (n = 9 per group) received 2, 5, 8, 11 and 14 Gy doses of TBI respectively, in a single-fraction irradiation. Physical doses within the chamber were assessed using model direct-reading dosimeters (Arrow-Tech, Inc. Rolla, ND, USA). The dose rate was 255 cGy/min in all treatment groups. Each treatment group was divided into three subgroups, for detailed investigation at 6, 24 and 72 h time points post-TBI respectively (n = 3).

## ^18^F-FDG-PET/CT imaging

The Tibet minipigs were scanned by ^18^F-FDG-PET/CT (Discovery-LS PET/CT scanner, GE Healthcare, Milwaukee, WI, USA) at the various observed time points. Certain measures were followed to ensure the objectivity and accuracy of the standard Uptake Value (SUV) and images [11, 12]: (i) All minipigs were required to fast for at least 8 h before undergoing imaging, and the serum glucose level was kept under 7–11 mmol/L; (ii) At 50–60 min after intravenous injection of the ^18^F-FDG (0.11–0.13 mCi/kg), a static whole-body emission PET/CT scan from head to pelvic floor was performed [15]; (iii) All minipigs received an intravenous injection of furosemide (0.5 mg/kg) and a 250 mL infusion of the saline solution, initiated 30 min after the ^18^F-FDG injection [16]; (iv) Using the fused PET/CT image, a region of interest (ROI, 3–4 cm^2^ in size) was placed in renal parenchyma. When a hypermetabolic SUV lesion was detected on the pre- and post-irradiation PET/CT images, the maximum SUV was respectively calculated using following equation: SUV = tissue concentration/injected FDG dose/body weight. If focal uptake was observed, an ROI was placed on the area of highest intensity determined visually. All of these images and data were processed by a Xeleris workstation system (GE, Healthcare).

### Histopathologic examination

The animals were humanely euthanized for serum and tissue collection. Euthanasia was carried out in accordance with the recommendations and guidelines of the American Veterinary Medical Association. Venous blood samples were collected from the minipigs for kidney function analysis and both kidneys were removed for histological observation at 6, 24 and 72 h post-irradiation. Both kidney samples were fixed in 10% phosphate-buffered formalin for 48 h, then the tissues were dehydrated in various grade of ethyl-alcohol and embedded in paraffin, three-micrometer sections were cut and stained with hematoxylin–eosin. To prepare the samples for examination by transmission electron microscopy (TEM), both kidneys were fixed in glutaraldehyde solution (2.5%), then fixed again in uranyl acetate (0.5%). Afterwards they were dehydrated and embedded in resin, cut into semi-thin and ultra-thin longitudinal sections and examined with a transmission electron microscope (JEOL 1010, Jeol, Tokyo, Japan).

### Blood sampling and processing

Venous blood was taken from minipigs ear vein and collected in normal tubes. Blood samples were left at 4 °C for 1 h, then centrifuged at 3500 rpm for 5 min to separate serum. All serum samples were transferred to the laboratory and were used for kidney function testing using Automatic Chemistry Analyzer (DH-1680, Nanfen Corp., Nanjing, China).

### Immunohistochemical staining

Immunohistochemistry (IHC) was carried out to assess IL-10 and TNF-α protein expression in kidney. Paraffin embedded kidney sections were deparaffinized, rehydrated, and then cooked in citrate buffer. Then sections were washed in blocking buffer [PBS with 1.0% bovine serum albumin (BSA)]. Antigen was retrieved using 1×-Tris–EDTA buffer (pH 9.0) for 15 min at 95 °C in pressure cooker. And then slides were incubated in 3% hydrogen peroxide for 10 min to block endogenous peroxidases. Primary antibody was incubated for 1 h at 1:50 dilution. Primary antibodies used in IHC, which were purchased from Abcam (Cambridge, UK), were rabbit monoclonal. Following this, all slides were washed with PBS and then incubated with secondary antibody that were conjugated with Streptavidin-peroxidase reagent for 10 min. Bound peroxidase activity was measured by a 3,3′diaminobenzidine (DAB substrate) and counterstaining was performed with hematoxylin.

### Western Blot

After treatment, the expression level of IL-10 and TNF-α in the kidney was detected. The extraction and quantitative analysis of total protein were performed according to the instructions of ProteoPrep^®^ Total Extraction Sample Kit (Sigma, USA). After SDS-PAGE electrophoresis, PVDF membrane transfer and antibody incubation were carried out in TBS buffer containing 5% skim milk, 1:500 dilution of IL-10 and TNF-α primary antibody respectively, and antibody binding took 2 h at room temperature. TBS was used to wash PVDF membrane for 5 min × 5 times before adding the secondary antibodies with 1:5000 dilution for another 1 h incubation at room temperature. Afterwards, the membrane was again washed for 5 min × 5 times with TBS before developing chemiluminescence and photographic fixing. Quantity One v4.4.0 was adopted for the optical density analysis of the corresponding bands of the target protein and the GAPDH.

### Statistical analysis

The data from the control and treatment groups were statistically analyzed by one-way analysis of variance (ANOVA) using a post hoc Tukey’s test for all pairwise multiple comparison procedures. The parameters were expressed mean ± SD. All data were processed by Statistical Product and Service Solutions (SPSS13.0, Guangzhou, China).

## Results

### SUV changes detected in both kidneys by PET at various radiation dosage and time points

**Fig. 1:**
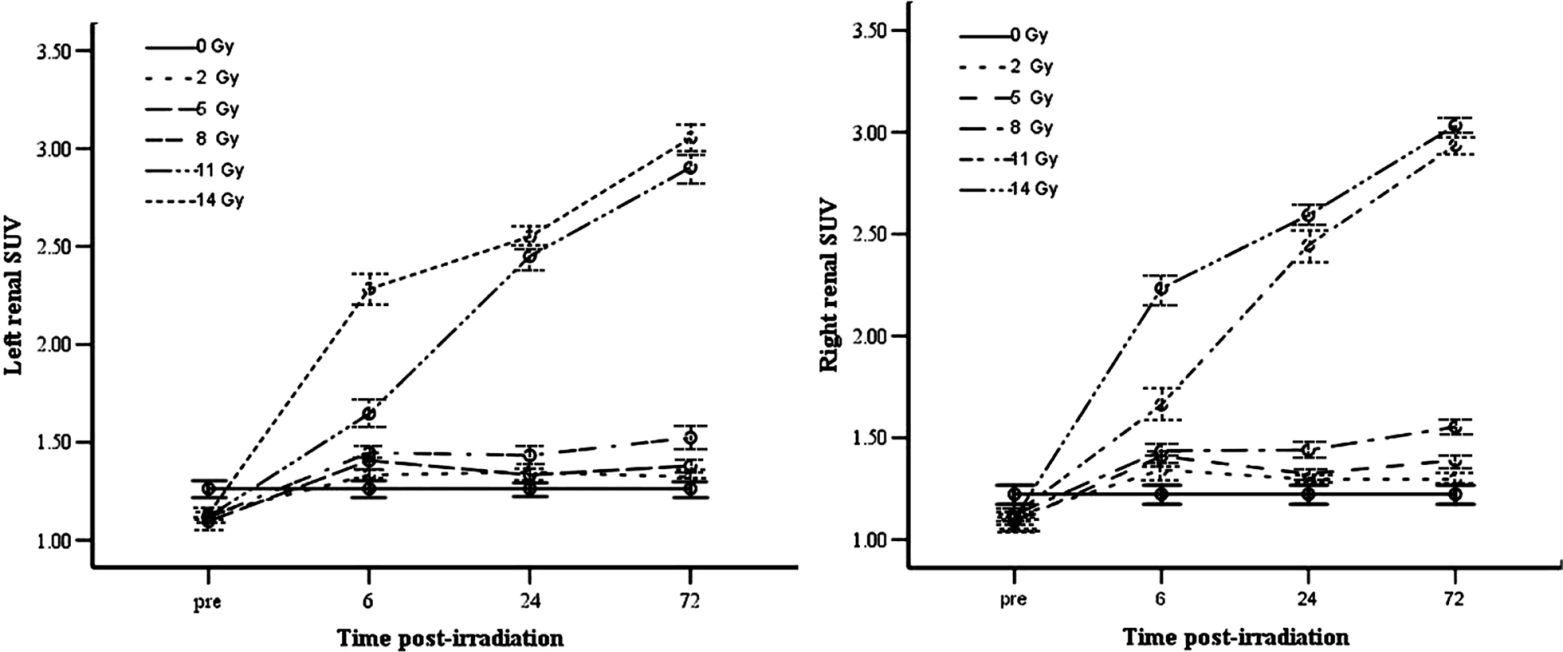
Comparison of kidney ^18^F-FDG uptake (SUV) change vs time at different TBI doses. In both kidneys, SUVs of both 11 and 14 Gy dose groups are significantly different in statistics from that of control group and presented an increase trend as time proceeded after TBI treatment. Both kidneys exhibited obvious interactive effect 72 h after 14 Gy TBI

All animals have survived the irradiation procedures and were maintained during the observation period after X-ray irradiation. As is shown in Fig. 1, time-dependent SUV change of both kidneys are apparently affected by radiation dosage. Compared to control group, SUVs of both 11 and 14 Gy dose groups are significantly different in statistics from that of control group (p < 0.05 and p < 0.01, respectively). Also notably, a highly significant difference in SUV (*p* < 0.01) was found between the 11 and 14 Gy dose groups at 6, 24 and 72 h post-radiation (there was no difference between left and right kidney *p *> 0.05, Table 1). At 24 h post-radiation, the average change in SUVs in the 11 and 14 Gy groups showed higher statistical difference (*p *< 0.01) compared with that in the control group. On another hand, for 2, 5 and 8 Gy dose groups, the SUVs didn’t show significant change in comparison to those in the control group (all *p* > 0.05) although there was obvious statistical significance in SUVs between the 8 and 11 Gy groups (*p *< 0.01). As time proceeded to 72 h post-radiation, the average changes in SUVs in the 8, 11 and 14 Gy dose groups were all significantly different (*p* < 0.01) as compared to that of the control group (*p* < 0.05, *p* < 0.01 and *p *< 0.01, respectively).

**Table 1:** The mean value of standard uptake value (SUV) of both kidneys

Dose (Gy)	Samples (n)	Pre	Time (h)			Total	F	P
		Left kidney	6	24	72			
Control	3	1.26 ± 0.05	1.26 ± 0.02	1.26 ± 0.02	1.26 ± 0.02	1.26 ± 0.01	0	1
2	9	1.13 ± 0.03	1.33 ± 0.05	1.34 ± 0.05	1.32 ± 0.04	1.28 ± 0.03	4.563	0.038
5	9	1.09 ± 0.02	1.40 ± 0.10	1.33 ± 0.06	1.38 ± 0.01	1.30 ± 0.04	4.901	0
8	9	1.11 ± 0.03	1.44 ± 0.08	1.43 ± 0.06	1.52 ± 0.02	1.38 ± 0.05	9.054	0.006
11	9	1.12 ± 0.05	1.64 ± 0.07	2.45 ± 0.09	2.90 ± 0.07	2.03 ± 0.21	139.804	0
14	9	1.13 ± 0.03	2.28 ± 0.09	2.55 ± 0.06	3.05 ± 0.06	2.25 ± 0.21	141.664	0
Total	48	1.14 ± 0.01	1.56 ± 0.08	1.72 ± 0.13	1.90 ± 0.18		195.21	0
t/F		2.675	22.91	105.019	301.972	225.289	F = 44.008	P = 0.000
P		0.075	0	0	0	0		

Dose (Gy)	Samples (n)	Pre	Time (h)			Total	F	P
		Right kidney	6	24	72			
Control	3	1.22 ± 0.02	1.22 ± 0.05	1.22 ± 0.05	1.22 ± 0.05	1.22 ± 0.02	0	1
2	9	1.11 ± 0.03	1.34 ± 0.07	1.29 ± 0.07	1.29 ± 1.08	1.26 ± 0.04	2.063	0
5	9	1.09 ± 0.03	1.41 ± 0.10	1.32 ± 0.07	1.39 ± 0.01	1.30 ± 0.04	5.86	0
8	9	1.12 ± 0.08	1.43 ± 0.11	1.44 ± 0.07	1.55 ± 0.02	1.38 ± 0.05	10.169	0
11	9	1.12 ± 0.06	1.66 ± 0.17	2.44 ± 0.07	2.93 ± 0.06	2.04 ± 0.21	100.68	0
14	9	1.10 ± 0.02	2.23 ± 0.07	2.59 ± 0.09	3.03 ± 0.04	2.24 ± 0.21	160.702	0
Total	48	1.12 ± 0.01	1.55 ± 0.08	1.72 ± 0.14	1.90 ± 0.18		188.081	0
t/F		1.063	19.418	72.71	230.713	114.942	F = 42.223	P = 0.00
P		0.427	0	0	0	0		

### Changes in PET/CT images and histopathological examination of both kidneys

**Fig. 2:**
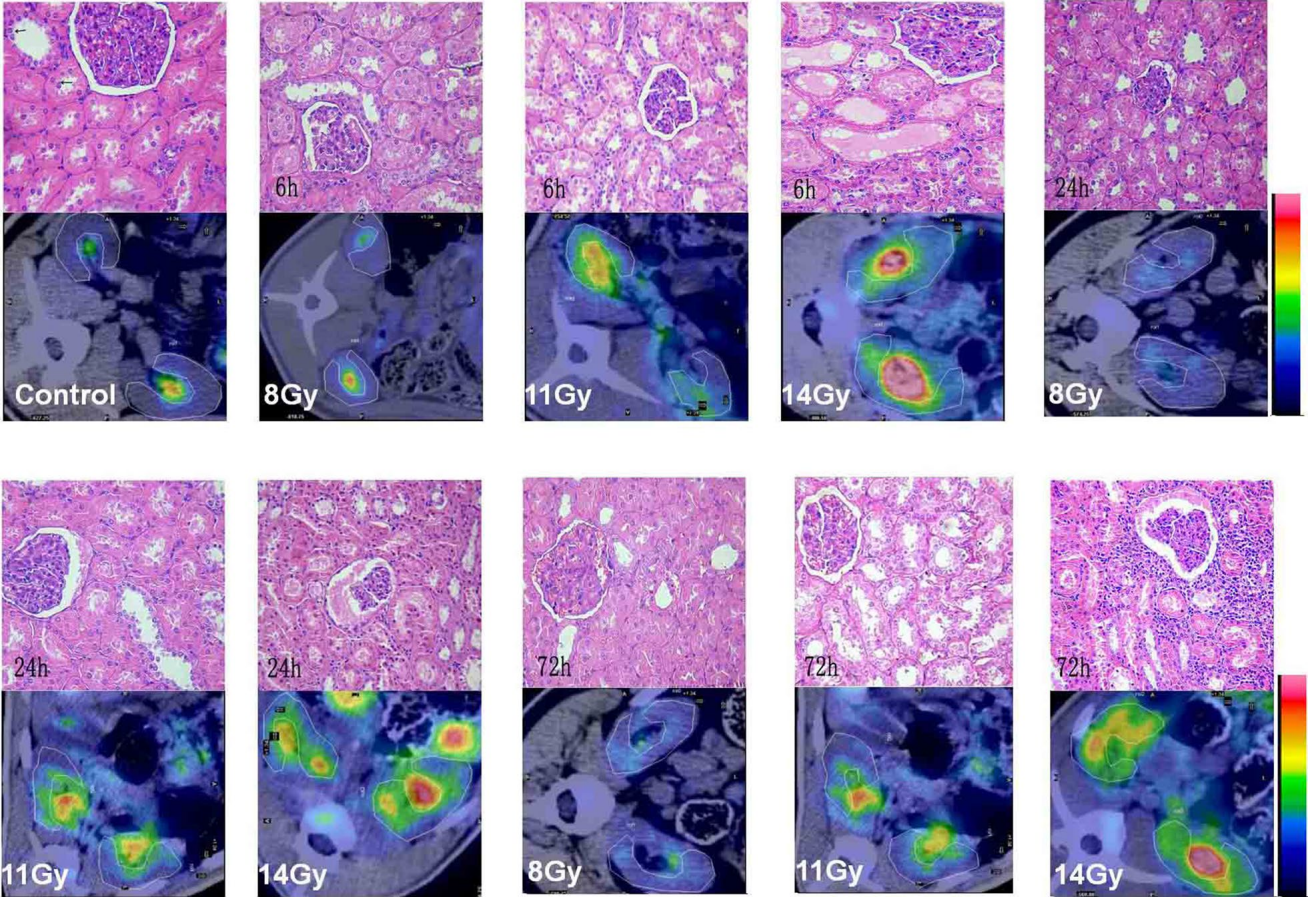
Changes in PET/CT images and histopathological examination of both kidneys after high TBI under microscope. Upper panel, the structure of normal renal tubule and glomeruli; degeneration of tubular cells was observed at dose of 8 Gy; formation of tube casts and degeneration and necrosis of tubular cells were observed at doses at dose of 11 Gy; formation of tube casts and degeneration and necrosis of tubular cells and the inflammatory cells infiltration of the renal interstitium were observed at dose of 14 Gy. Bottom panel. For normal both-kidneys ^18^F-FDG uptake was diffusive and generally of a low level; ^18^F-FDG uptake was generally of a low level and exhibited an inhomogeneous distribution at dose of 8 Gy irradiation; ^18^F-FDG uptake was generally of a high level and exhibited an inhomogeneous distribution at dose of 11 Gy irradiation; ^18^F-FDG uptake was generally of a higher level and exhibited an inhomogeneous distribution at dose of 14 Gy irradiation (H&E stain, original magnification 10 × 20)

In histopathological analysis of both kidneys, a highly significant difference can be discerned between the 11 and 14 Gy dose groups at 6, 24 and 72 h post-radiation. The dynamic PET/CT imaging revealed the pattern and kinetics of ^18^F-FDG distribution (Fig. 2). Accompanying time points’ increase for the 14 Gy dose group, the value of ^18^F-FDG uptake increased more markedly in the bilateral renal parenchyma, comparable to the values obtained at 24 and 72 h in the 11 Gy dose group (very high ^18^F-FDG uptake was found around the renal pelvis and calices and are themselves metabolic product). However, FDG uptake in the 2, 5, 8 Gy dose groups are low, which is similar to that in control group. Under light microscopy, for 11 Gy irradiation treatment, we observed the formation of tube casts degeneration and necrosis of tubular cells, while for 14 Gy irradiation treatment we observed the formation of tube casts, degeneration and necrosis of tubular cells, and inflammatory cell infiltration in renal interstitium (Fig. 2).

### Ultrastructural changes observed in both kidneys at different dosages and time points after TBI

**Fig. 3:**
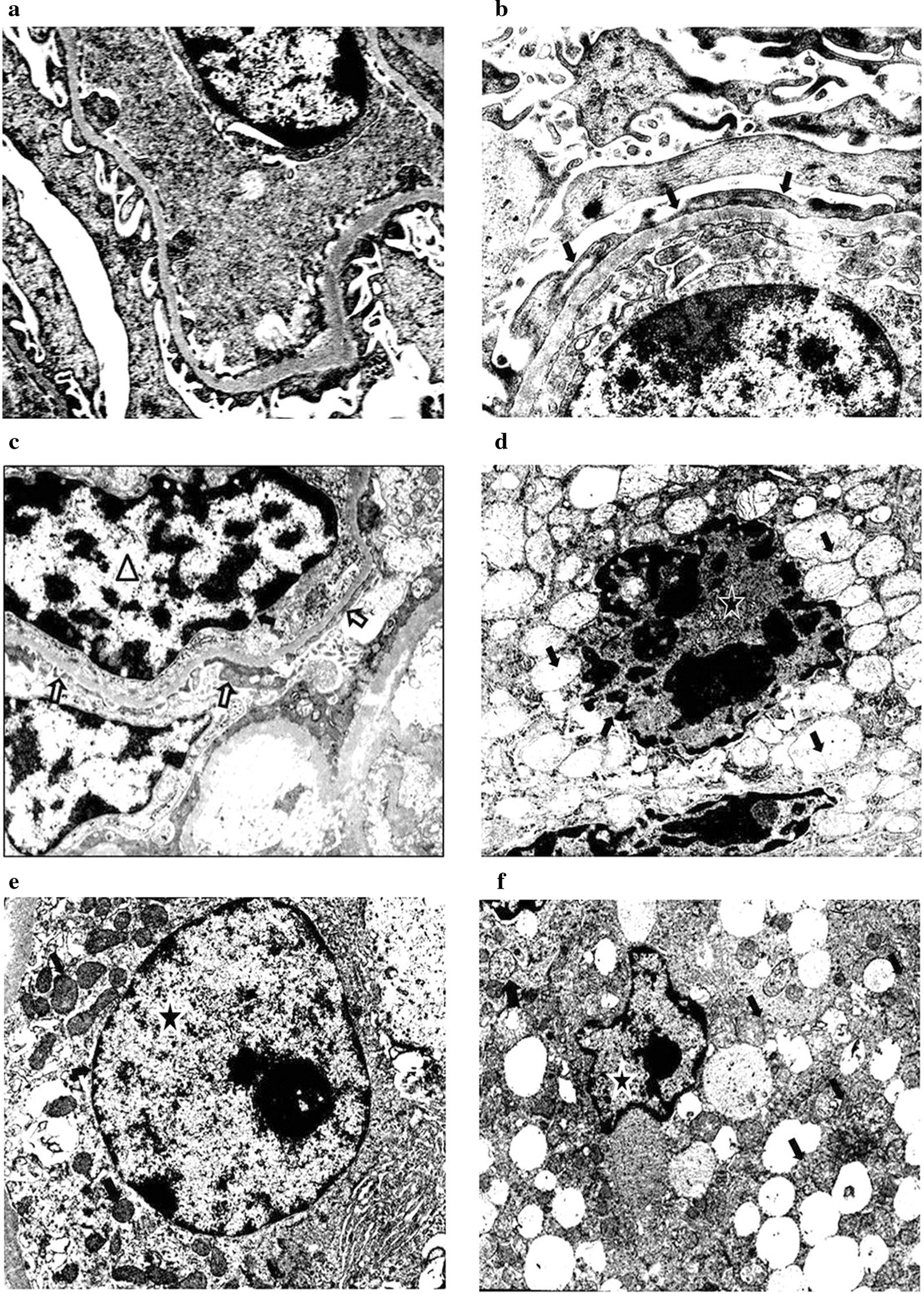
The scanning electron microscope examination of both kidneys after TBI. **a** Degeneration and necrosis of tubular cells (arrows) and inflammatory cell infiltration of the renal interstitium (triangle) were observed 24 h after TBI at dose of 14 Gy. **b** Formation of tube casts and the degeneration and necrosis of tubular cells (arrows) were observed 6 h after TBI at dose of 14 Gy. (H&E stain, original magnification 10 × 20). **c** There were overlying epithelial cell foot processes in the control group (arrows). **d** 6 h after TBI at dose of 11 Gy, the overlying epithelial cell foot processes were effaced and ran together (arrows); Also observed were the chromatin condensation and swelling of the endothelial cells (triangle). **e** Normal structure of renal tubule epithelial cells in the control group (pentagon). **f** 6 h after TBI at dose of 8 Gy, there were pyknotic nuclei, chromatin condensation of renal tubule epithelial cells (pentagon), dilatation of the mitochondria, numerous lysosomal structures, and lipid droplets (arrows). (Magnification: ×10,000)

Most animals in the 2, 5 and 8 Gy groups showed no significant gross morphologic changes in the experiment. Areas of congestion and petechial hemorrhages were noted in the 11 and 14 Gy groups. Definite abnormalities were showing up at 24 h under light microscopy, especially in the 14 Gy dose group (Fig. 3a, b). We observed acute congestion, inflammatory cell infiltration in renal interstitium, formation of tube casts and the degeneration and necrosis of tubular cells. However, no obvious glomerular changes were noted except for congestion and endotheliocytic swelling of vessels. In contrast to the findings described above, under electron microscopy distinct changes were visible at 6 h in the 11 Gy dose group (Fig. 3c, d). We observed obvious heterochromatin of the glomerular epithelial cells, and endotheliocytic and focal fusion of foot processes. This became more marked with increase in observation time and dosage. The tubules manifested focal degenerative changes in the cytoplasm and nucleus as early as 6 h following 8 Gy irradiation (Fig. 3e, f), including chromatin condensation and aggregation, dilatation of the agranular endoplasmic reticulum and the mitochondria, numerous lysosomal structures, and lipid droplets.

### Renal functional tests at various TBI dosage and time points

**Fig. 4:**
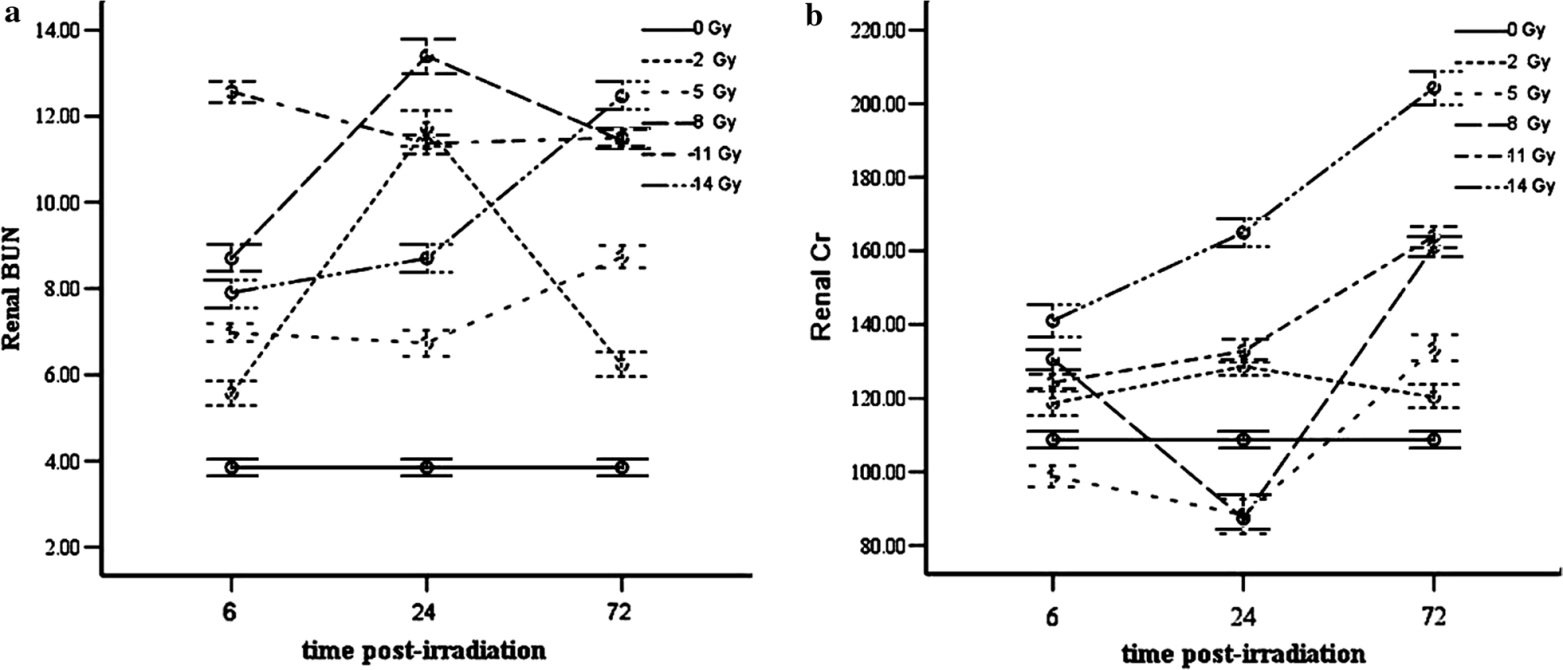
Renal functional tests at various TBI dosage and time points. **a** BUN values of TBI treatment groups as compared to the control group; each bar represents the mean ± SD of the BUN. **b** Cr values of treatment group as compared to the control group; each bar represents the mean ± SD of the Cr (post hoc Turkey test was adopted as the pairwise multiple comparison procedure)

Kidney function tests mainly involve measurement of BUN and Cr. As is shown in Fig. 4a, compared with the control group, at 6 h post-radiation, there was an obvious significant difference in BUN values between the 8, 11 and 14 Gy dose groups (*p* < 0.05, *p* < 0.05, *p *< 0.01); at 24 h post-radiation, the values in the 8 Gy dose group were higher than those observed in the other two groups (*p *< 0.01); at 72 h post-radiation, there was a significant statistical difference between the 8 and 14 Gy group (*p* < 0.05). When comparing BUN values change vs time in each treatment group, the values corresponding to three time points in the 14 Gy group were significantly different among each other (*p* < 0.01).

To analyze Cr data (Fig. 4b), at the earliest observation time points (6 h post-radiation), the Cr value for the 14 Gy group was higher than those for the 8 Gy group (*p* < 0.01); at 24 h post-radiation, the Cr value for the 14 Gy group was of significant statistical difference as compared to other groups (*p* < 0.01); at 72 h post-radiation, the relevant values are of significant difference between the 11 and 14 Gy groups (*p* < 0.01). When comparing Cr values’ change vs time in each treatment group, the values corresponding to three time points in the 11 Gy group and 14 Gy group respectively were significantly different among each other inside each group (*p* < 0.01). There was also statistical difference between the different time points in the other treatment groups, but no regular change pattern was presented as those in 11/14 Gy dose groups, whose Cr value change is positively correlated with post-TBI time increase.

### Expression of IL-10 and TNF-α in the kidney after TBI injury

**Fig. 5:**
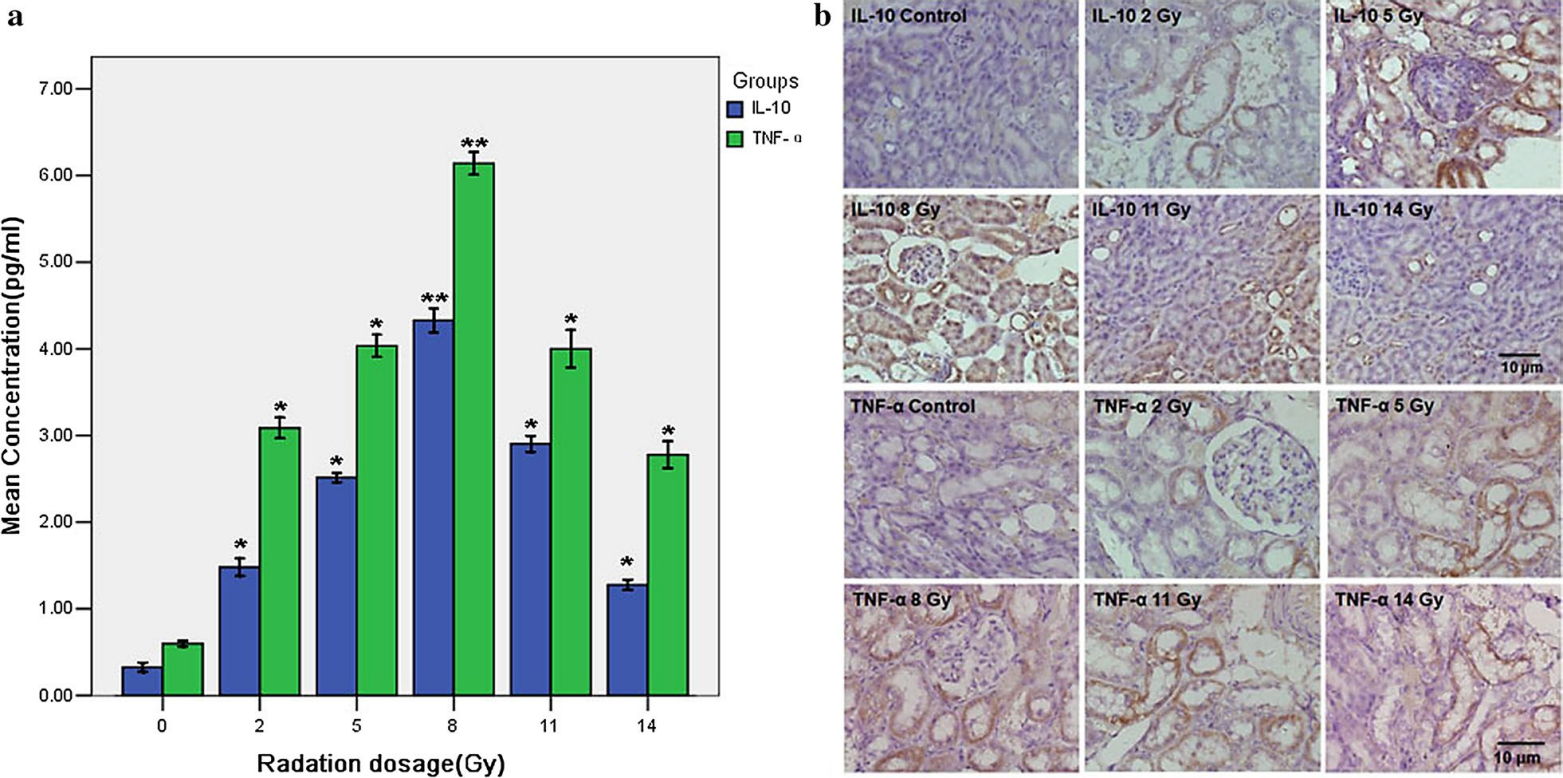
Expression of IL-10 and TNF-α in the kidney after TBI injury. The kidney tissues were harvested from TBI-treated Tibet minipigs at doses of 2, 5, 8, 11 and 14 Gy respectively, fixed in formalin for 1 day, and then embedded with paraffin. **a** Levels of IL-10 and TNF-α in kidney tissue of TBI injury minipigs. The cytokine levels in kidney tissue of TBI-treated Tibet minipigs at doses of 2, 5, 8, 11 and 14 Gy were assessed. The concentrations of IL-10 and TNF-α were the highest at dose of 8 Gy. **b** Immunohistochemical staining for IL-10 and TNF-α were performed by anti-IL-10 antibody and anti-TNF-α antibody respectively. Positive staining for IL-10 and TNF-α were distributed abundantly along the renal tubule and collecting duct. The sham kidney exhibited faint staining for IL-10, whereas TBI caused the strongest staining at 8 Gy. Data are presented as mean ± SEM. **p < 0.01; *p < 0.05 vs the sham kidney (magnification ×400)

**Fig. 6:**
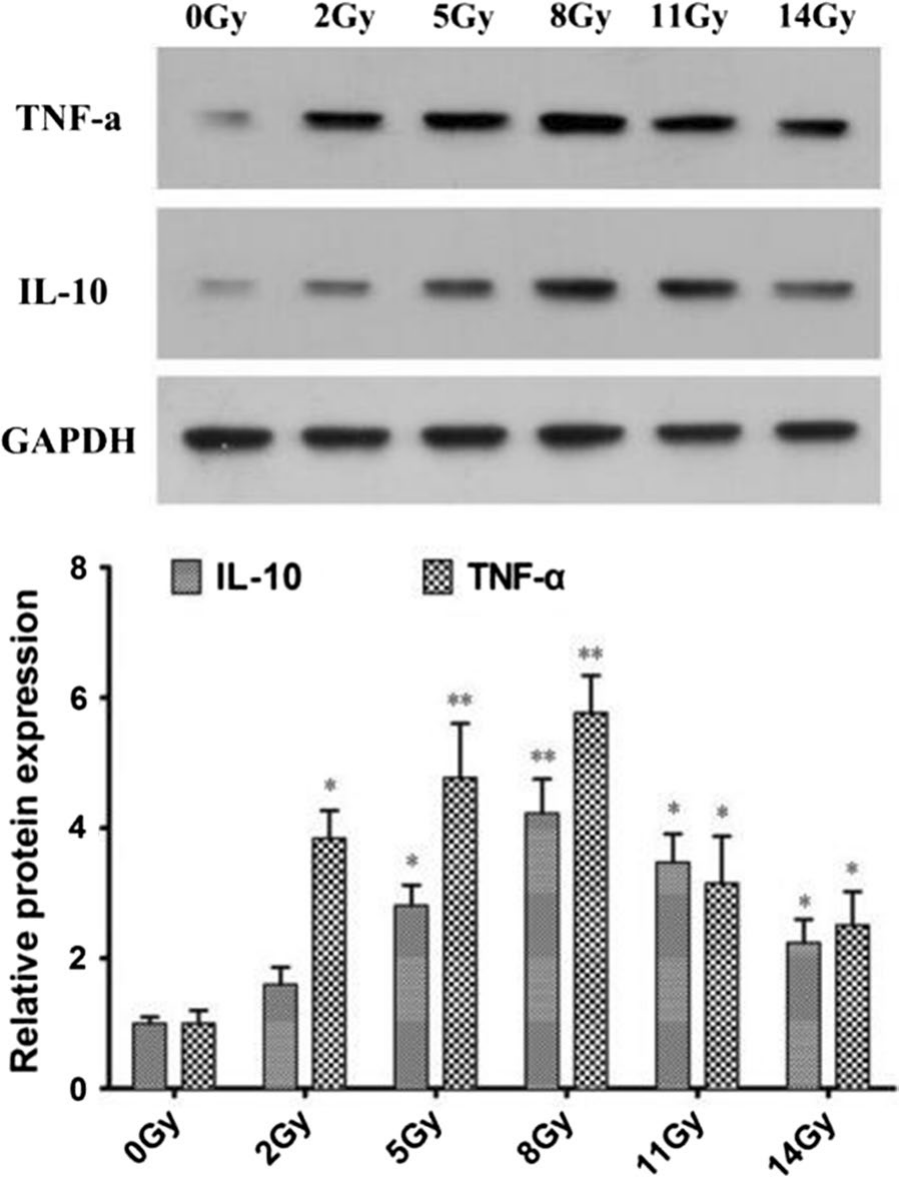
The expression of IL-10 and TNF-α in the kidney were detected by Western Blot after TBI injury. The expression levels of IL-10 and TNF-α can been induced by TBI injury in kidney tissue of Tibet minipigs. The concentrations of IL-10 and TNF-α were the highest in kidneys treated at dose of 8 Gy. Data are presented as mean ± SEM. *p < 0.01; *p < 0.05 vs the sham kidney

To examine the localization of IL-10 and TNF-α, immunohistochemical staining studies were performed. The positive fluorescence staining for IL-10 and TNF-α was distributed abundantly along the renal tubule and collecting duct, which was observed initially at 2 Gy treatment and reached peak values at 8 Gy treatment (Fig. 5). Western Blot assay were used to determine expression proteins for IL-10 and TNF-α in kidney tissues of control and dose groups treated by 2 Gy, 5 Gy, 8 Gy, 11 Gy and 14 Gy radiation respectively, using specific antibodies for IL-10 and TNF-α. We found markedly increased IL-10 and TNF-α protein expression levels after TBI dosed at 2 Gy and 5 Gy, which reached a peak at 8 Gy and then decreased at 11 Gy and 14 Gy as demonstrated in Fig. 6. The results are consistent with immunohistochemical staining analyses.

## Discussion

No research has previously been conducted on the application of PET/CT in detecting radiation-induced kidney damage (RIKD). ^18^F-FDG is a structural analogue of 2-deoxyglucose and so is likely to be enriched in tissues upon a high glucose consumption. Activated inflammatory cells also show increased expression of glucose transporters, resulting in ^18^F-FDG accumulation [13]. In our study, comparing PET values pre- and post-radiation exposure, the results showed that PET/CT, can be used as a diagnosis imaging method to detect RIKD in Tibet minipigs. In addition, the results of ^18^F-FDG PET/CT were consistent with other test results (especially the results of BUN concentrations). Further research is now needed to determine whether this method can be applied to humans, since in humans FDG accumulates to a significant degree in the urinary systems including the renal pelvis due to rapid urine excretion, and the anatomic structure of kidneys of Tibet minipigs is not totally identical to that of humans. Our findings also highlighted that the SUVs may be related to dosages and observed time intervals. Generally, uptake of ^18^F-FDG depends upon glucose transporters expressed in the cell membrane, the local cell density, and the metabolic activity of the surrounding tissue [14]. Histological microscopy observations from earlier studies have shown that tubules are more susceptible to radiation than glomerular tissue [15], and concentrative glucose transporters have been identified in the apical domain of the proximal tubules of the kidney. On the other hand, sugars molecules are mainly metabolized in the proximal tubules. Therefore, the uptake of ^18^F-FDG may be dependent on glucose transporters expressed in the proximal tubules. Moreover, activated inflammatory cells in renal interstitium increase the expression of glucose transporters resulting in ^18^F-FDG accumulation [13]. The results were possibly caused by the increase of activated inflammatory cells in renal tubule and collecting duct. The expression levels of IL-10 and TNF-α protein were positively correlated with radiation doses up to 8 Gy.

The mechanism of radiation damage on the kidney has not yet been clarified. Compared with other structural elements of the kidney, some authors consider that tubules are the most susceptible to radiation [8], whereas others hold a different opinion [9]. Our findings are based on a single large dose of radiation and are consistent with the previous results. The proximal convoluted tubules showed degenerative changes in the cytoplasm that were visible under a light microscope as early as 6 h after 11 Gy irradiation. Damage evident in the 8 Gy groups under an electron microscope included chromatin condensation and aggregation, dilatation of the mitochondria, numerous lysosomal structures, lipid droplets and fusion of foot processes. These changes are similar to those occurred in case of mild nephrosis, except that the glomerular tissue showed no obvious damage. Over the past few decades the lowest dose needed to cause kidney damage has been fiercely debated by researchers. Sarin et al. reported that the tolerance dose of whole kidneys after TBI is 14 Gy in humans [16], whereas, Safwat et al. stated that 11 Gy is the lowest dose to cause kidneys damage in a mouse model [17]. Our findings showed that the lowest dose to cause damage visible under microscopy is between 8 and 11 Gy in the Tibet minipig model.

## Conclusion

In conclusion, our study showed that ^18^F-FDG PET/CT can reflect pathological changes in kidneys and it may be a useful tool for rapid and non-invasive assessment in cases of suspected radiation-induced kidney damage.
